# Association of immune checkpoint inhibitors therapy with arterial thromboembolic events in cancer patients: A retrospective cohort study

**DOI:** 10.1002/cam4.6455

**Published:** 2023-08-16

**Authors:** Jie Zhu, Yue Chen, Yuanlong Zhang, Wei Wang, Yujue Wang, Zhuo Lu, Yulin Zhang, Haike Lei, Dairong Li, Bo Long, Haixia Liu

**Affiliations:** ^1^ Department of Cardio‐Oncology Chongqing University Cancer Hospital Chongqing China; ^2^ School of Medicine Chongqing University Chongqing China; ^3^ Medical Record Management Department Chongqing University Cancer Hospital Chongqing China; ^4^ Chongqing Cancer Multi‐Omics Big Data Application Engineering Research Center Chongqing University Cancer Hospital Chongqing China; ^5^ Department of Medical Oncology Chongqing University Cancer Hospital Chongqing China

**Keywords:** acute coronary syndrome, arterial thromboembolism events, immune checkpoint inhibitors, peripheral arterial thromboembolism, retrospective cohort study, stroke, transient ischemic attack

## Abstract

**Background:**

Immune checkpoint inhibitors (ICIs) have emerged as a standard treatment for various malignancies. However, research indicates blocking the immune checkpoint pathway may exacerbate atherosclerotic lesions.

**Objectives:**

We aimed to investigate whether ICI therapy increases the risk of arterial thromboembolic events (ATEs).

**Methods:**

A retrospective cohort study was conducted on patients with histologically confirmed cancer at our institution between 2018 and 2021, using the propensity score matching method. The primary endpoint was ATEs occurrence, comprising acute coronary syndrome, stroke/transient ischemic attack, and peripheral arterial thromboembolism. Subgroup analyses assessed whether the ICI treatment effect on ATEs varied over time by limiting the maximum follow‐up duration. Logistic regression analysis identified ATE risk factors in ICI‐treated patients.

**Results:**

Overall, the ICI group (*n* = 2877) demonstrated an ATEs risk 2.01 times higher than the non‐ICI group (RR, 2.01 [95% CI (1.61–2.51)]; *p* < 0.001). Subgroup analysis revealed no significant increase in ATEs risk for ICI‐treated patients within 1 year (Limited to a max 9‐month follow‐up, *p* = 0.075). However, ATEs risk in the ICI group rose by 41% at 1 year (*p* = 0.010) and 97% at 4 years (*p* ≤ 0.001). Age, diabetes, hypertension, peripheral atherosclerosis, atrial fibrillation, chronic ischemic heart disease, distant cancer metastasis, and ICI treatment cycles contributed to ATEs risk elevation in ICI‐treated patients.

**Conclusion:**

ICI‐treated patients may exhibit a higher risk of ATEs, especially after 1 year of treatment.

## Graphical abstract

Notably, our study reveals a novel insight that Immune checkpoint inhibitor (ICI) treatment does not increase the risk of arterial thromboembolic events (ATEs) within the first year, but the risk significantly increases after 1 year of treatment and becomes more prominent as the follow‐up duration extends. Beyond the established traditional risk factors (older age, diabetes, hypertension, PAAS, AF, and CIHD) for ATEs, distant cancer metastasis and an increasing number of ICI cycles are emerging risk factors for ATEs in ICI‐treated patients.

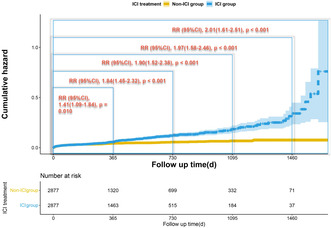

## 1 INTRODUCTION

Significant advancements in the diagnosis and treatment of malignancies have dramatically extended the survival time of cancer patients. Over the past few years, immune checkpoint inhibitors (ICIs) have revolutionized cancer therapeutics, representing a milestone in oncology. By binding to immune checkpoints, ICI therapy restores and enhances the ability of effector T cells to recognize and kill tumor cells, thereby augmenting the antitumor immune response. Currently, the clinical utilization of ICI agents primarily involves inhibitors targeting the programmed cell death protein 1 (PD‐1), programmed death ligand 1 (PD‐L1), cytotoxic T lymphocyte‐associated antigen 4 (CTLA‐4), and lymphocyte‐activation gene 3 (LAG‐3) pathways.

ICI therapy has demonstrated remarkable efficacy in both solid and hematological tumors. Presently, nine checkpoint inhibitors targeting the PD‐1/PD‐L1, CTLA‐4, and LAG‐3 pathways have been approved by the United States Food and Drug Administration for the treatment of subsets of patients with 17 different types of cancer, such as head and neck cancer, breast cancer, lung cancer, liver cancer, esophageal cancer, stomach cancer, colorectal cancer, kidney cancer, bladder cancer, uterine (endometrial) cancer, cervical cancer, Merkel cell carcinoma, skin squamous cell carcinoma, basal cell carcinoma, melanoma, lymphoma, and mesothelioma, as of May 2023.^1^ Furthermore, an increasing number of indications for ICI therapy are currently under investigation. In the United States, it is estimated that 43.63% of cancer patients are eligible for ICI treatment in 2018.^2^

Nevertheless, the therapeutic blockade of the immune checkpoint pathway by ICI disrupts the balance between immunity and tolerance in the immune system. Consequently, the activated immune system may attack other nontumor tissues, causing immune‐related adverse events (IRAEs). Studies have reported that up to 94.9% of cancer patients treated with ICIs experience at least one IRAE, with 55.5% encountering at least one severe IRAE (grades 3 and 4).^3^ Moreover, immune checkpoints serve as negative regulators of atherosclerosis and play a crucial role in its formation.^4^ Atherosclerosis is a well‐known cause of arterial thromboembolic events (ATEs), such as acute myocardial infarction and stroke, which can lead to an increased risk of death and significantly impact subsequent anticancer treatment. A few retrospective clinical studies have recently examined the relationship between ICI treatment and ATEs, yet their conclusions remain inconsistent.^5^, ^6^

Considering the widespread clinical use of ICI therapy and its potential impact on the incidence of ATEs in patients with malignant tumors, we designed a propensity score matching (PSM) cohort study to determine whether the administration of ICIs results in an increase in ATEs.

## 2 METHODS

### 2.1 Study design and population

**FIGURE 1:**
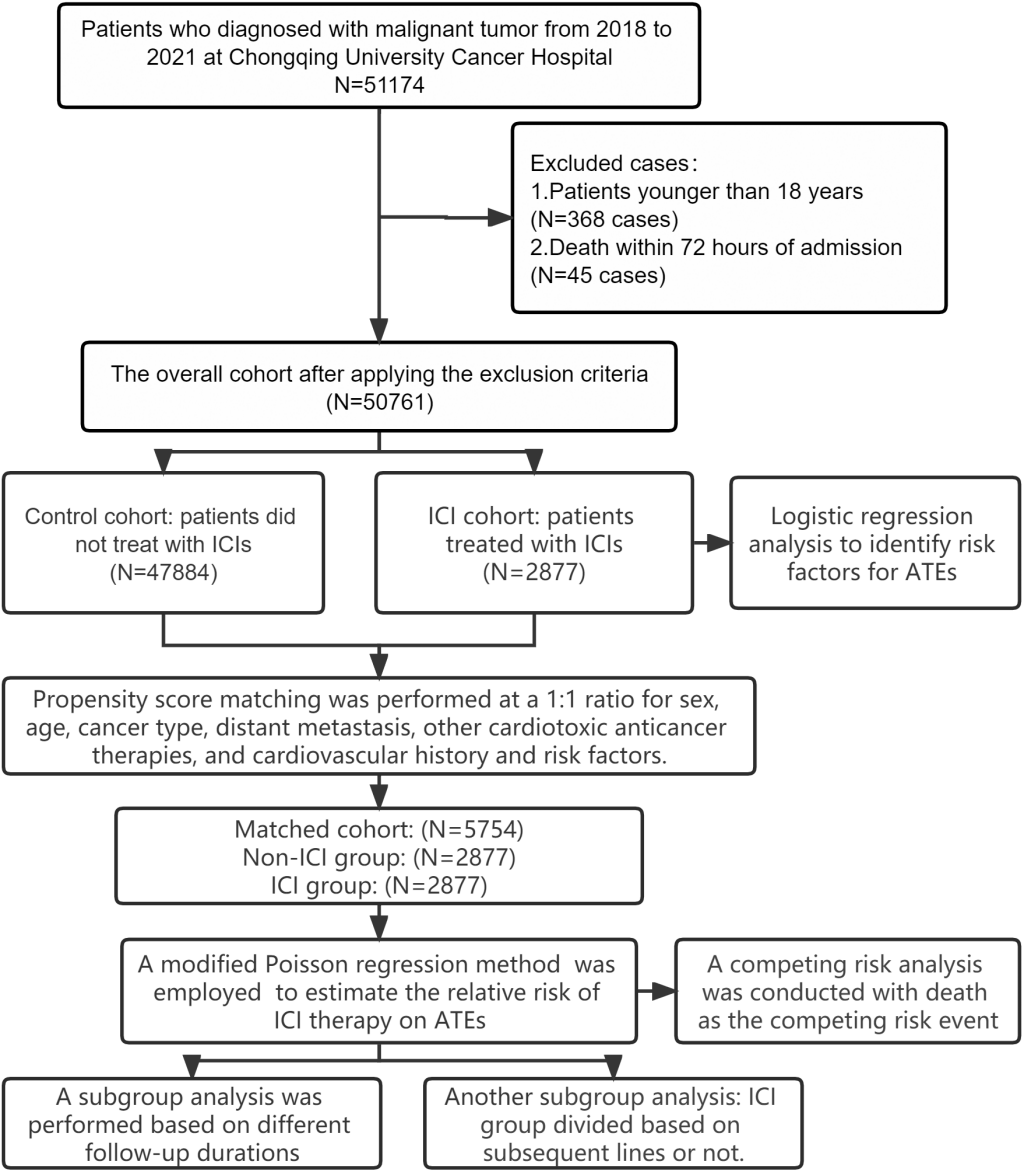
Flowchart of the study design and patients selection process. ATEs, arterial thromboembolic events; ICIs, immune checkpoint inhibitors.

We conducted a retrospective cohort study to investigate the association between ICI treatment and the incidence of ATEs in cancer patients. We included patients diagnosed with malignant tumors at Chongqing University Cancer Hospital between 2018 and 2021. The exclusion criteria were^1^ patients younger than 18 years of age and^2^ patients who died within 72 h after admission. All patients treated with ICIs were included in the ICI cohort. Patients who did not receive ICI therapy were assigned to the control cohort. The ICI and control cohorts were matched using the PSM method at a 1:1 ratio based on sex, age, cancer type and stage, other cardiotoxic anticancer therapies, and history of cardiovascular diseases and risk factors. All research procedures are summarized in Figure 1. This study was conducted in accordance with the Helsinki Declaration and was approved by the Ethics Committee of our research institute. Due to the retrospective cohort design of our study, the Ethics Committee granted a waiver of informed consent from the patients. The study design also adhered to the items listed in the STROBE (STrengthening the Reporting of OBservational studies in Epidemiology) guidelines^7^ for cohort studies to ensure the consistency and quality of the study. Patients were followed up until September 30, 2022.

### 2.2 Ascertainment of clinical outcome

The study's primary outcome was the occurrence of ATEs, which encompassed ischemic stroke, transient ischemic attack (TIA), acute coronary syndrome (ACS), and peripheral arterial thrombosis and embolism (PATE), including thromboembolism of the renal, mesenteric, and splenic arteries. The secondary endpoints were the individual components of the ATEs. We verified endpoint event occurrences by retrieving the corresponding International Statistical Classification of Diseases and Related Health Problems, Tenth Revision (ICD‐10) codes from the medical records of hospitalized patients at our institution. Specifically, the ACS codes used were I20.0, I20.1, I21, I22, and I23, while codes for stroke were I63.0‐I63.6 and I63.9. TIA was represented by the code G45.900. The codes for PATE were I74.0–I74.5, I74.8, and I74.9, and thromboembolisms of the mesenteric, renal, and splenic arteries were coded as K55.003, N28.0, and D73.500, respectively. Specific endpoint events ICD‐10 codes are summarized in Tables [Link], [Link].

### 2.3 Data collection

Three researchers independently collected data using preestablished forms to reduce selection bias through our cancer center's big data platform for digital medical records. One investigator collected baseline demographic data (age, sex), cardiovascular history (hypertension, chronic ischemic heart disease, myocardial infarction, stroke, peripheral arterial atherosclerosis, heart failure [HF]), atrial flutter or atrial fibrillation (AF), and cardiovascular risk factors (diabetes mellitus, hyperlipidemia, chronic kidney disease). Another researcher gathered cancer‐related data (such as cancer types, distant metastasis, and cardiotoxic anticancer therapies). All cardiovascular histories, cardiovascular risk factors, and cancer types involved in disease diagnoses were retrieved and verified using ICD‐10 codes. Distant metastasis of cancer was confirmed through detailed TNM(tumor (T), node (N), and metastasis (M)) staging. A third investigator performed data collection of the primary and secondary outcomes. Consultations with a fourth investigator resolved disputes over the data collection procedure.

### 2.4 Propensity score matching process

To mitigate selection bias and obtain more accurate causal effect estimates, propensity score matching (PSM) was employed using the “MatchIt” package in R software (R Core Team, 2021). Initially, a binomial distribution model with a generalized linear model was fitted to calculate the propensity scores for each observed unit. The model was based on the relationship between the treatment variable (e.g., ICIs) and potential confounders (e.g., demographic data, cardiovascular history, cardiovascular risk factors, cancer types, distant cancer metastasis, cardiotoxic anticancer therapies) that could influence treatment assignment and outcomes. This enabled the prediction of the probability of each observed unit receiving the treatment given their covariates. Subsequently, the “matchit()” function from the “MatchIt” package in R was applied to perform matching based on the propensity scores.^8^

### 2.5 Statistical analysis

Patient demographics and clinical characteristics were assessed using descriptive statistics. Categorical variables were summarized using counts and percentages and analyzed with the chi‐square test. Mean and standard deviation were presented for continuous variables. Descriptive statistics were utilized to assess the distribution of patient demographics and clinical characteristics. Counts and percentages were used to summarize categorical variables, which were then analyzed using the chi‐square test. Mean and standard deviation were presented for continuous variables. Independent sample t‐tests and corrected *t*‐tests were applied to examine normally distributed and nonnormally distributed measurements, respectively. The relative risk (RR) and 95% confidence interval (CI) for the primary and secondary outcomes in the matched cohort were estimated using a modified Poisson regression method^9^ (Poisson regression with robust error variance) through Stata Statistical Software/Special Edition 12.0 (StataCorp LP., T.X., USA). A subgroup analysis was conducted by restricting the maximum follow‐up time to half a year, 9 months, 1, 2, 3, and 4 years, to investigate whether the impact of ICI treatment on ATEs risk varied with follow‐up time. Another subgroup analysis was conducted to divide the ICI group into two subgroups based on whether ICI treatment was administered as subsequent lines or not. This analysis aimed to examine the impact of ICIs as different lines of treatment on ATEs. Kaplan–Meier‐based cumulative hazard curves for the primary outcome were generated using the “survival” and “survminer” packages in R. Logistic regression analysis was performed to calculate the odds ratio (OR) and 95% CI for all baseline characteristics in the ICI cohort using the built‐in “glm()” function in R. Only the first event was counted if a patient experienced two or more endpoint events. Variables with a *p* value less than 0.10 in the univariate logistic analysis were included in the multivariate analysis. Considering the high mortality rate among cancer patients, which may introduce competing risks for endpoint events, we conducted a competing risk analysis with death as a competing risk event. For univariate analysis, the Fine‐Gray test was employed. All statistical tests were two‐tailed, and *p* values less than 0.05 were deemed statistically significant.

## 3 RESULTS

### 3.1 Baseline characteristics

At our institution, from January 1, 2018 to December 31, 2021, a total of 51,174 patients were diagnosed with malignant tumors. The baseline characteristics of these patients are summarized in Table 1. After applying the exclusion criteria, a total of 50,761 patients were studied (excluding 368 patients younger than 18 and 45 patients who died within 72 h after admission). The ICI cohort included 2877 patients, with 934 (32.5%) being women. The mean age was 58.22 (±10.98) years, and the mean follow‐up time was 441.77 (±345.69) days. In the ICI cohort, 2411 (83.8%) and 430 (14.9%) patients received PD‐1 and PD‐L1 monoclonal antibody monotherapy, respectively, while 36 (1.2%) patients received both. The average ICI use was 4.73 (±5.44) cycles. The top three cancers in the ICI cohort were lung cancer, liver cancer, and esophageal cancer, accounting for 40.3%, 11.5%, and 10.9% of patients, respectively. Distant organ metastasis occurred in 66.9% of patients treated with ICIs. In terms of baseline cardiovascular history and risk factors, 16.4% of patients had hypertension, 16.3% had hyperlipidemia, and 10.3% had diabetes. For cardiotoxicity anticancer treatments, 42.9% of cancer patients received radiotherapy, and chemotherapy was dominated by platinum and paclitaxel, accounting for 59.2% and 37.1% of patients, respectively. There were no significant differences at baseline between the two groups after PSM, except for the rate of peripheral arterial atherosclerosis.

**TABLE 1:** Baseline characteristics of the overall cohort and matched cohort.

Variables	Overall cohort	Control cohort	ICI cohort	*p*	Matched cohort	Non‐ICI group	ICI group	*p*
	50,761	47,884	2877		5754	2877	2877	
Age, mean (SD)	58.71 (12.67)	58.74 (12.77)	58.22 (10.98)	0.033	58.25 (11.25)	58.28 (11.51)	58.22 (10.98)	0.846
Sex—female, *n* (%)	24,574 (48.4)	23,640 (49.4)	934 (32.5)	<0.001	1877 (32.6)	943 (32.8)	934 (32.5)	0.822
Follow up (days), mean (SD)	220.60 (311.19)	207.31 (303.92)	441.77 (345.69)	<0.001	444.86 (391.95)	447.96 (433.33)	441.77 (345.69)	0.549
Cardiovascular history and risk factors, *n* (%)								
Hypertension	7753 (15.3)	7282 (15.2)	471 (16.4)	0.097	949 (16.5)	478 (16.6)	471 (16.4)	0.831
Diabetes	4419 (8.7)	4122 (8.6)	297 (10.3)	0.002	603 (10.5)	306 (10.6)	297 (10.3)	0.731
Hyperlipidemia	3421 (6.7)	2953 (6.2)	468 (16.3)	<0.001	882 (15.3)	414 (14.4)	468 (16.3)	0.052
PAAS	3203 (6.3)	2905 (6.1)	298 (10.4)	<0.001	546 (9.5)	248 (8.6)	298 (10.4)	0.028
CIHD	2215 (4.4)	2078 (4.3)	137 (4.8)	0.303	260 (4.5)	123 (4.3)	137 (4.8)	0.409
HF	1120 (2.2)	1064 (2.2)	56 (1.9)	0.362	95 (1.7)	39 (1.4)	56 (1.9)	0.098
CKD	740 (1.5)	634 (1.3)	106 (3.7)	<0.001	195 (3.4)	89 (3.1)	106 (3.7)	0.244
AF	505 (1.0)	469 (1.0)	36 (1.3)	0.183	65 (1.1)	29 (1.0)	36 (1.3)	0.454
History of stroke	263 (0.5)	248 (0.5)	15 (0.5)	1	33 (0.6)	18 (0.6)	15 (0.5)	0.727
History of MI	41 (0.1)	41 (0.1)	0 (0.0)	0.218	0 (0.0)	0 (0.0)	0 (0.0)	NA*
Cancer types and distant metastasis, *n* (%)								
Distant metastasis	19,635 (38.7)	17,711 (37.0)	1924 (66.9)	<0.001	3862 (67.1)	1938 (67.4)	1924 (66.9)	0.715
Lung cancer	13,593 (26.8)	12,435 (26.0)	1158 (40.3)	<0.001	2276 (39.6)	1118 (38.9)	1158 (40.3)	0.293
Metrocarcinoma	5344 (10.5)	5077 (10.6)	267 (9.3)	0.027	542 (9.4)	275 (9.6)	267 (9.3)	0.752
Colorectal cancer	4086 (8.0)	3978 (8.3)	108 (3.8)	<0.001	221 (3.8)	113 (3.9)	108 (3.8)	0.784
Liver cancer	2799 (5.5)	2468 (5.2)	331 (11.5)	<0.001	666 (11.6)	335 (11.6)	331 (11.5)	0.902
Esophageal cancer	2601 (5.1)	2286 (4.8)	315 (10.9)	<0.001	633 (11.0)	318 (11.1)	315 (10.9)	0.933
Lymphoma	1899 (3.7)	1840 (3.8)	59 (2.1)	<0.001	120 (2.1)	61 (2.1)	59 (2.1)	0.926
Gastric cancer	1727 (3.4)	1674 (3.5)	53 (1.8)	<0.001	114 (2.0)	61 (2.1)	53 (1.8)	0.508
NPC	1604 (3.2)	1457 (3.0)	147 (5.1)	<0.001	273 (4.7)	126 (4.4)	147 (5.1)	0.215
Ovarian cancer	1303 (2.6)	1251 (2.6)	52 (1.8)	0.01	112 (1.9)	60 (2.1)	52 (1.8)	0.504
Urologic malignancy	1262 (2.5)	1160 (2.4)	102 (3.5)	<0.001	207 (3.6)	105 (3.6)	102 (3.5)	0.887
MNEPL	507 (1.0)	478 (1.0)	29 (1.0)	1	59 (1.0)	30 (1.0)	29 (1.0)	1
CMM	329 (0.6)	263 (0.5)	66 (2.3)	<0.001	136 (2.4)	70 (2.4)	66 (2.3)	0.795
Other cancers	13,707 (27.0)	13,517 (28.2)	190 (6.6)	<0.001	395 (6.9)	205 (7.1)	190 (6.6)	0.465
Other treatment, n (%)								
Grade 3 or 4 surgery	18,210 (35.9)	17,264 (36.1)	946 (32.9)	0.001	1865 (32.4)	919 (31.9)	946 (32.9)	0.464
Platinum	12,893 (25.4)	11,189 (23.4)	1704 (59.2)	<0.001	3381 (58.8)	1677 (58.3)	1704 (59.2)	0.486
Paclitaxel	7972 (15.7)	6905 (14.4)	1067 (37.1)	<0.001	2117 (36.8)	1050 (36.5)	1067 (37.1)	0.662
Cyclophosphamide	3321 (6.5)	3284 (6.9)	37 (1.3)	<0.001	68 (1.2)	31 (1.1)	37 (1.3)	0.542
Fluorouracil	3252 (6.4)	2840 (5.9)	412 (14.3)	<0.001	808 (14.0)	396 (13.8)	412 (14.3)	0.569
Bevacizumab	1011 (2.0)	843 (1.8)	168 (5.8)	<0.001	349 (6.1)	181 (6.3)	168 (5.8)	0.507
Radiotherapy	11,903 (23.4)	10,668 (22.3)	1235 (42.9)	<0.001	2478 (43.1)	1243 (43.2)	1235 (42.9)	0.852

*Note*: NA* = There were no patients in the ICI cohort who with p had a history of MI.

Abbreviations: AF, atrial fibrillation or atrial flutter; CIHD, chronic ischemic heart disease; CKD, chronic kidney disease; CMM, cutaneous malignant melanoma; HF, heart failure; ICI, immune checkpoint inhibitor; MI, myocardial infarction; MNEPL, malignant tumors of nasal cavity, middle ear, paranasal sinuses and larynx; NPC, nasopharyngeal carcinoma; PAAS, peripheral arterial atherosclerosis; SD, standard deviation.

### 3.2 Primary and secondary outcomes

The overall incidence of ATEs was 3.4% among the cohort of 50,761 individuals. In contrast, the incidence of ATEs was 7.8% in the subset of patients who received ICI treatment. The risk of ATEs in the ICI group was 2.01 times higher than that in the non‐ICI group (RR, 2.01 [95% CI (1.61–2.01)]; *p* < 0.001) in matched cohort. The competing risk analysis, with death as a competing risk event, demonstrated that even when accounting for competing risk events, the cumulative risk of ATEs in the ICI group remained significantly higher than that in the non‐ICI group (*p* < 0.001). Figure S1 displays the results of the competing risk analysis. For the secondary outcomes, compared with the non‐ICI group, the risk of Stroke/TIA in the ICI group increased by 78% (RR, 1.78 [95% CI (1.32–2.41)]; *p* < 0.001), whereas the risk of ACS and PATE in the ICI group increased by 1.70 times (RR, 2.70 [95% CI (1.62–4.50)]; *p* < 0.001) and 1.36 times (RR, 2.36 [95% CI (1.19–2.77)]; *p* = 0.006), respectively.

### 3.3 Subgroup analysis

**FIGURE 2:**
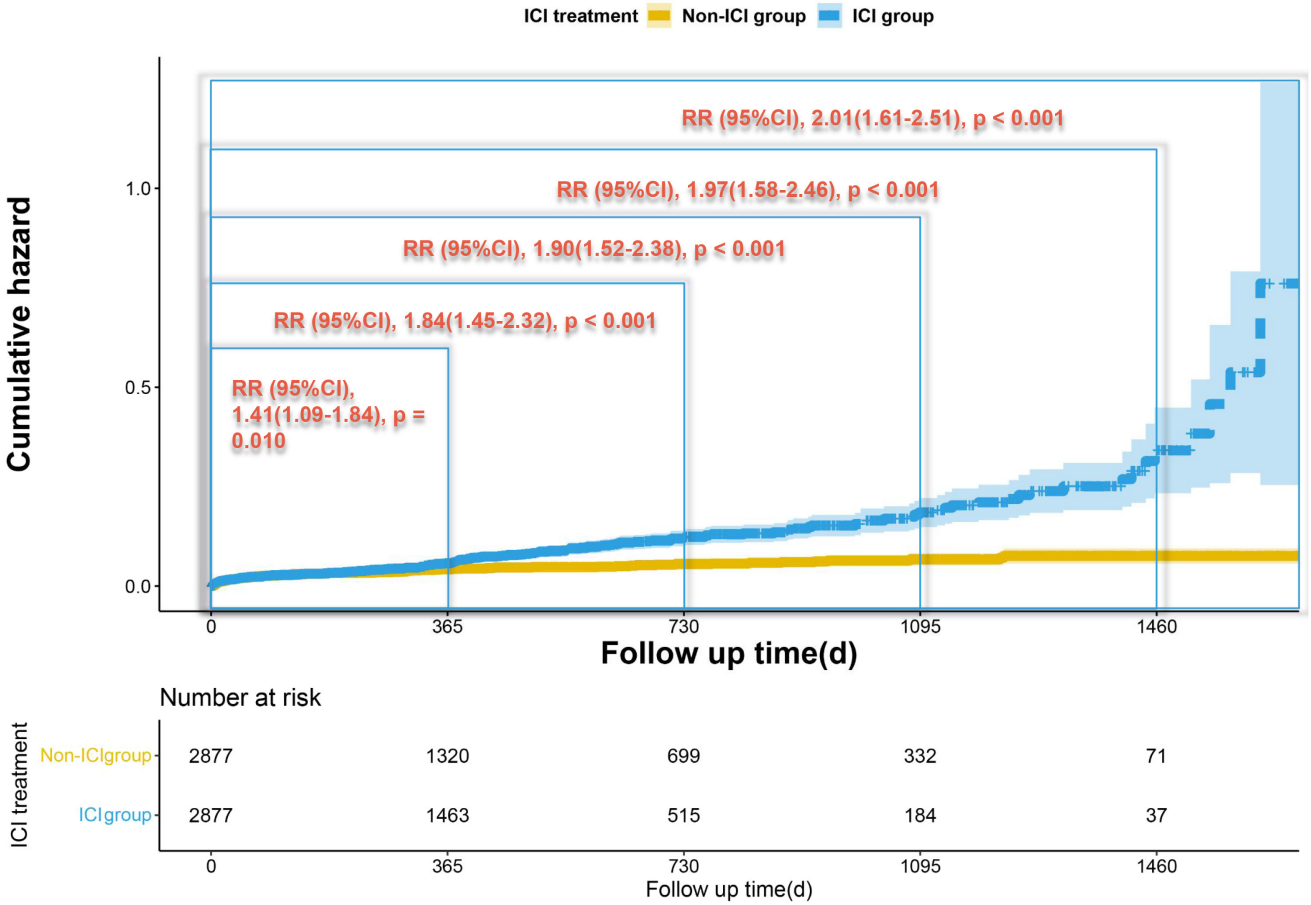
Kaplan–Meier‐based cumulative hazard curves of the primary outcome over different maximum follow‐up duration. Patients receiving immune checkpoint inhibitors for malignancies exhibited a significantly heightened risk of arterial thromboembolic events at 1 year and beyond, with the risk becoming more prominent as the follow‐up duration extended. The *p* value comes from modified Poisson regression. RR, relative risk; CI, confidence interval.

The subgroup analysis showed that ICI treatment did not significantly increase the incidence of ATEs in cancer patients within 1 year (6‐month follow‐up: RR, 1.10 [95% CI (0.81–1.50)], *p* = 0.52; 9‐month follow‐up: RR, 1.30 [95% CI (0.97–1.72)], *p* = 0.075). However, the risk of ATEs in the ICI group increased significantly at 1 year (RR, 1.41 [95% CI (1.09–1.84)]; *p* = 0.010) and became increasingly evident with the extension of the follow‐up duration (2‐year follow‐up: RR, 1.84 [95% CI (1.45–2.32)], *p* ≤ 0.001; 3‐year follow‐up: RR, 1.90 [95% CI (1.52–2.38)], *p* ≤ 0.001; 4‐year follow‐up: RR, 1.97 [95% CI (1.58–2.46)], *p* ≤ 0.001). Table 2 presents the primary outcome of the matched cohort at different follow‐up times, whereas Figure 2 displays the cumulative hazard curves of ATEs at varying follow‐up times after ICI treatment. Moreover, in comparison with the non‐ICI group, the risk of ATEs was significantly higher in both the cohort receiving ICI treatment only (RR, 1.92 [95% CI (1.31–2.82)]), *p* < 0.001) and the cohort receiving ICIs as subsequent lines (RR, 2.02 [95% CI (1.61–2.54)]), *p* < 0.001). The results are displayed in Figures S2 and S3.

**TABLE 2:** The primary outcome over different follow‐up times in the matched cohort.

Follow‐up	RR (95% CI)	*p* * value
6 months	1.10 (0.81–1.50)	0.52
9 months	1.30 (0.97–1.72)	0.075
1 year	1.41 (1.09–1.84)	0.010
2 years	1.84 (1.45–2.32)	<0.001
3 years	1.90 (1.52–2.38)	<0.001
4 years	1.97 (1.58–2.46)	<0.001

Abbreviations: CI, confidence interval; RR, relative risk.

* *p* value comes from modified Poisson regression.

### 3.4 Risk factors for ATEs in the ICI cohort

The baseline characteristics with a P value less than 0.10 in the univariate logistic regression analysis in the ICI cohort were included in the multivariate analysis. The multivariate analysis showed that age (OR, 1.02 [95% CI (1.00–1.04)]; *p* = 0.045), diabetes mellitus (OR, 6.03 [95% CI (4.28–8.48)]; *p* < 0.001), hypertension (OR, 4.50 [95% CI (3.23–6.28)]; *p* < 0.001), peripheral arterial atherosclerosis (OR, 2.99 [95% CI (2.02–4.39)]; *p* < 0.001), AF (OR, 2.72 [95% CI (1.08–6.37)]; *p* = 0.026), chronic ischemic heart disease (OR, 1.72 [95% CI (1.02–2.85)]; *p* = 0.040), distant metastasis of malignant tumor (OR, 2.03 [95% CI (1.38–3.04)]; *p* < 0.001), and cycles of ICIs use (OR, 1.04 [95% CI (1.01–1.07)]; *p* = 0.002) were independent risk factors for ATEs in cancer patients treated with ICIs. Table 3 summarizes the results of the logistic regression analysis.

**TABLE 3:** Summary of univariate and multivariate logistic regression analysis results in ICI cohort.

Variables^a^	Univariate logistic regression, OR (95% CI)	*p*	Multivariate logistic regression, OR (95% CI)	*p*
Age	1.06 (1.04–1.07)	<0.001	1.02 (1.00–1.04)	0.045
Sex—female	0.64 (0.46–0.87)	0.005	1.05 (0.70–1.56)	0.807
Hypertension	7.65 (5.76–10.19)	<0.001	4.50 (3.23–6.28)	<0.001
Diabetes	9.29 (6.87–12.55)	<0.001	6.03 (4.28–8.48)	<0.001
Hyperlipidemia	1.53 (1.09–2.11)	0.012	0.88 (0.58–1.30)	0.521
HF	4.12 (2.14–7.49)	<0.001	1.61 (0.70–3.51)	0.247
AF	4.70 (2.13–9.58)	<0.001	2.72 (1.08–6.37)	0.026
PAAS	4.00 (2.89–5.49)	<0.001	2.99 (2.02–4.39)	<0.001
CIHD	5.70 (3.79–8.42)	<0.001	1.72 (1.02–2.85)	0.040
History of stroke	4.35 (1.20–12.82)	0.012	1.40 (0.32–5.07)	0.628
Distant metastasis	2.35 (1.68–3.37)	<0.001	2.03 (1.38–3.04)	<0.001
Lung cancer	2.04 (1.55–2.69)	<0.001	1.19 (0.82–1.72)	0.362
Liver cancer	0.61 (0.35–0.98)	0.055	1.11 (0.58–2.04)	0.740
Metrocarcinoma	0.48 (0.24–0.85)	0.021	0.81 (0.36–1.72)	0.596
NPC	0.32 (0.10–0.76)	0.025	0.33 (0.09–0.91)	0.052
Paclitaxel	1.31 (0.10–1.73)	0.052	1.36 (0.97–1.91)	0.070
ICI cycles	1.02 (1.00–1.05)	0.037	1.04 (1.01–1.07)	0.002

Abbreviations: AF, atrial fibrillation or atrial flutter; CI, confidence interval; CIHD, chronic ischemic heart disease; HF, heart failure; ICIs, immune checkpoint inhibitors; NPC, nasopharyngeal carcinoma; OR, odds ratio; PAAS, peripheral arterial atherosclerosis.

^a^ Only variables with a *p* value of <0.1 in univariate logistic regression analysis are listed in the table.

## 4 DISCUSSION

The present study was a large‐scale, real‐world retrospective cohort study focusing on the association between ATEs and ICI treatment. Our study had two main conclusions. First, our research has unveiled the novel discovery that ICI treatment does not increase ATEs in cancer patients within 1 year, but the risk significantly increases after 1 year and becomes more pronounced with increased follow‐up. Second, the risk factors for ATEs in cancer patients treated with ICIs were age, diabetes mellitus, hypertension, peripheral arterial atherosclerosis, AF, chronic ischemic heart disease, distant metastasis of malignant tumors, and cycles of ICIs use.

Immune checkpoint molecules are expressed on immune cells and can regulate immune activation, playing a critical role in preventing autoimmune responses. Abnormal expression or function of immune checkpoint molecules can lead to various diseases. For instance, excessive expression of immune checkpoint molecules can increase the risk of malignant tumors by inhibiting immune function. Conversely, defects or low expression of immune checkpoint molecules can cause autoimmune diseases by causing the body's immune response to go out of control. Monoclonal antibody drugs targeting CTLA‐4, PD‐1, and PD‐L1 can regulate the immune response, exerting anticancer effects. Thus, ICI agents are considered broad‐spectrum anticancer drugs that have shown remarkable clinical efficacy in treating various types of cancer. As the indications of ICIs rapidly expand and the treatment gradually switches from second‐line to first‐line, ICIs may become the primary treatment for various types of cancer in the near future, as well as aspirin or statins for cardiovascular diseases. Unfortunately, ICI agents have a variety of unique adverse reactions related to their mechanism of action, which are called IRAEs. Clinicians have gradually emphasized the importance of ICI cardiovascular adverse events after continuing reports of ICI‐related severe myocarditis.^10^, ^11^

The most meaningful finding of this study was that the hazard of ATEs increased significantly in cancer patients treated with ICIs after 1 year and became more pronounced with increased follow‐up. Previous preclinical studies have shown that immune checkpoints have a protective effect on atherosclerosis. Abatacept, a syngeneic analog of CTLA‐4, prevented CD4+ T‐cell activation and reduced atherosclerosis progression by 78% in the femoral artery of mice.^12^ Similarly, atherosclerotic plaque size in mice with elevated homocysteine levels was associated with reduced CTLA‐4 membrane expression, while abatacept pretreatment improved plaque development and decreased macrophage content.^13^ One study has shown that PD‐1, in combination with its ligand PD‐L1/PD‐L2, plays an arterial protective role by inhibiting the production of Th1 cytokines and promoting Treg cell development.^14^ The use of ICIs to block the binding of immune checkpoints to their ligands could lead to the progression and deterioration of atherosclerotic plaques. A trial conducted by Poels et al. showed that mice treated with CTLA inhibitors had a twofold increase in the size of atherosclerotic lesions and more advanced plaque phenotypes (reduced collagen content, thickened intima, and increased necrotic core), which was mainly mediated by a transition to an activated T‐cell profile in plaques.^15^ Moreover, both PD‐L1 deficiency and PD‐1 inhibition in hyperlipidemic mice were associated with increased atherosclerotic lesions and elevated T‐cell activation in plaques.^16^, ^17^ In addition, a study demonstrated that PD‐1 was highly expressed in T cells within human atherosclerotic plaques at baseline.^18^ However, PD‐1 and PD‐L1 expression decreased in circulating T cells and myeloid dendritic cells in patients with ACS or coronary heart disease.^19^, ^20^

A large retrospective clinical study matched 2842 cancer patients treated with ICIs with patients not treated with ICIs at a 1:1 ratio based on age, cancer type, and cardiovascular history. After more than 2 years of follow‐up, the study found that ICI treatment was associated with an over 3‐fold progression of aortic plaques; more importantly, using ICIs increased the risk of complex cardiovascular events (myocardial infarction, coronary revascularization, and ischemic stroke) by more than fourfold.^21^ The results of our study were consistent with those of previous studies showing that ICI treatment significantly increased the ATEs risk in cancer patients. The analysis of secondary outcomes revealed that the incidences of stroke/TIA, ACS, and PATE were all increased to contrasting degrees. This confirmed the fact that ICI therapy has a systemic effect on atherosclerotic plaques. ICI treatment could exacerbate the progression of plaque lesions, lead to the instability of plaques and ultimately increase the risk of ATEs.

Additionally, our research showed that the risk of ATEs in the ICI group increased significantly after 1 year and became increasingly evident with the extension of the follow‐up duration. The reason might be that atherosclerosis is a long‐term, progressive process. Atherosclerosis is a chronic inflammatory disease that can be quantified using positron emission tomography (PET) imaging to assess the degree of inflammation.^22^ The most commonly used PET tracer for atherosclerosis imaging is 18F‐fluorodeoxyglucose (FDG).^23^ In a study of 10 melanoma patients who received short‐term ICI treatment, there was no increase in 18F‐FDG uptake in the chest aorta and neck arteries before and after treatment, indicating that short‐term ICI treatment did not affect vascular inflammation in melanoma patients.^24^ Animal experiments from the same study demonstrated that ICI treatment did not affect marrow‐driven vascular inflammation in hyperlipidemic mice. However, ICIs' inhibition of CTLA‐4 and PD‐1 exacerbated T‐cell‐mediated inflammation in atherosclerotic plaques, thereby worsening plaque progression to clinically unfavorable phenotypes.^24^ This may be attributed to the chronic process of human atherosclerotic plaque formation. Similarly, ICIs' effect on human atherosclerotic plaque through T‐cell‐mediated inflammation is a chronic process that takes a long time to cause unstable plaque and increase the risk of ATEs. Therefore, our research found that ICIs significantly increased the risk of ATEs only after 1 year and beyond, and this effect became more pronounced with longer follow‐up times. Further extensive research is required to clarify the long‐term effects of immune checkpoint inhibition on human atherosclerosis.

A pharmacovigilance study (using the VigiBase database) showed that the incidence of myocardial infarction and cerebral arterial ischemia in cancer patients treated with ICIs was only 0.53% and 0.62%, respectively, which was even lower than the data reported in the complete database.^5^ A meta‐analysis of clinical trials of PD‐1 and PD‐L1 inhibitors in treating nonsmall‐cell lung cancer found a 1% incidence of myocardial infarction and a 2% incidence of stroke.^25^ However, recent retrospective studies have found that the incidence of ATEs in patients treated with ICIs ranges from 4.2% to 10.3%, far exceeding the data reported in previous phase II–III clinical trials. Drobni et al. reported an incidence of a composite endpoint of myocardial infarction, coronary revascularization, and ischemic stroke of 4.2% in 2842 ICI‐treated cancer patients.^21^ In a single‐center study conducted by Oren et al., 3326 patients with malignancy treated with ICIs had a 7% rate of myocardial infarction and stroke over 16 months.^26^ A nationwide study in Denmark showed that 743 lung cancer and 145 melanoma patients treated with ICIs had 1‐year absolute composite outcome (myocarditis, pericarditis, HF, cardiovascular death, tachyarrhythmia, and bradyarrhythmia) risks of 9.7% and 6.6%, respectively.^27^ Laenens et al. even reported an incidence of major adverse cardiovascular events (including acute coronary syndrome, stroke/TIA, and HF) of 10.3% in 672 ICI‐treated cancer patients during a mean follow‐up of 13 months.^6^ The results of our study were consistent with those of previous retrospective cohort studies, which suggested that the incidence of ATEs was 7.8% in a cohort of 2877 ICI‐treated patients with an average follow‐up of 441.77 days.

The incidence of ATEs was reported to be extremely low in preclinical ICI drug trials, but recent retrospective studies found a significant increase in ATEs. One of the prominent reasons was that early clinical drug trials excluded patients with atherosclerotic cardiovascular disease; however, a significant portion of patients who received ICI therapy in the real world had a variety of atherosclerotic cardiovascular diseases. For instance, Drobni et al. found that approximately 20.5% of cancer patients with ICI therapy had a history of atherosclerotic cardiovascular disease.^21^ Our study also revealed that a significant percentage of the patients treated with ICIs had cardiovascular diseases or risk factors, such as hypertension (accounting for 16.4%), hyperlipidemia (16.3%), peripheral arterial atherosclerosis (10.4%), diabetes (10.3%), and chronic ischemic heart disease (4.8%). Furthermore, inadequate follow‐up time during clinical drug experiments of ICIs may have contributed to the low incidence of ATEs observed in these trials. Our research findings are consistent with the fact that the risk of ATEs significantly increases approximately 1 year after ICI treatment. Multivariate logistic regression analysis detected that age, diabetes mellitus, hypertension, peripheral arterial atherosclerosis, AF, and chronic ischemic heart disease were high‐risk factors for ATEs in cancer patients treated with ICIs. In addition, we found that distant metastasis of malignant tumors and the cycles of ICIs use were also associated with ATEs.

## 5 STUDY LIMITATIONS

First, although we performed PSM for demographic characteristics, baseline cardiovascular risk factors, cancer types, and other types of cardiotoxic anticancer therapy, there may be additional unmeasured confounding factors influencing the risk of ATEs in cancer patients. Our database, for example, lacked data on smoking, obesity, antiplatelet, anticoagulant, and anti‐atherosclerosis treatments. In addition, our study showed that 66.9% of patients treated with ICIs had distant organ metastasis, and these patients might have poor adherence to anti‐thrombotic and anti‐atherosclerosis therapy due to expected short survival, high risk of bleeding, and liver and kidney dysfunction. This could potentially overestimate the incidence of ATEs. Second, our study was based on information in the medical records of hospitalized patients at our institution; the true incidence of ATEs might be underestimated if cancer patients had ATEs and subsequently visited another institution for management. Third, this was a single‐center study, which might lead to regional bias.

## 6 CONCLUSIONS

There is a higher risk of ATEs in patients undergoing ICI treatment, and the risk increases significantly after 1 year of treatment. Close monitoring of ATEs is recommended during and after ICI therapy for cancer patients who have hypertension, peripheral arterial atherosclerosis, AF, chronic ischemic heart disease, older age, diabetes, distant metastases, and an increased number of ICI therapy cycles.

## AUTHOR CONTRIBUTIONS

**Jie Zhu:** Conceptualization (equal); data curation (equal); investigation (equal); methodology (equal); project administration (equal); software (equal); validation (equal); writing – original draft (lead); writing – review and editing (equal). **Yue Chen:** Conceptualization (equal); data curation (equal); formal analysis (equal); methodology (equal); software (equal); writing – original draft (equal); writing – review and editing (equal). **Yuanlong Zhang:** Conceptualization (equal); data curation (equal); formal analysis (equal); funding acquisition (equal); methodology (equal); project administration (equal); software (equal); writing – original draft (equal); writing – review and editing (equal). **Wei Wang:** Conceptualization (equal); data curation (equal); methodology (equal); software (equal); writing – review and editing (equal). **Yujue Wang:** Conceptualization (equal); data curation (equal); methodology (equal); project administration (equal); visualization (equal); writing – review and editing (equal). **Zhuo Lu:** Conceptualization (equal); data curation (equal); investigation (equal); methodology (equal); writing – review and editing (equal). **Yulin Zhang:** Conceptualization (equal); data curation (equal); methodology (equal); project administration (equal); supervision (equal); writing – review and editing (equal). **Haike Lei:** Conceptualization (equal); data curation (equal); methodology (equal); project administration (equal); supervision (equal); writing – review and editing (equal). **Dairong Li:** Conceptualization (equal); formal analysis (equal); investigation (equal); methodology (equal); project administration (equal); supervision (equal); writing – review and editing (equal). **Bo Long:** Conceptualization (equal); funding acquisition (equal); methodology (equal); project administration (equal); supervision (equal); writing – review and editing (equal). **Haixia Liu:** Conceptualization (equal); funding acquisition (equal); investigation (equal); methodology (equal); project administration (equal); supervision (equal); writing – original draft (equal); writing – review and editing (equal).

## FUNDING INFORMATION

This research received support from a decision‐making consultation and management innovation project in the Shapingba District of Chongqing, China (grant no. Jcd202276). and funded by the Chongqing Natural Science Foundation (grant no. CSTB2022NSCQ‐MSX1482). These funding sources are unrelated to the work submitted.

## CONFLICT OF INTEREST STATEMENT

The authors declare that they have no potential conflict of interest.

## ETHICS APPROVAL AND CONSENT TO PARTICIPATE

This study was conducted in accordance with the Helsinki Declaration and was approved by the Ethics Committee of Chongqing University Cancer Hospital (CZLS2023021‐A). Due to the retrospective cohort design of our study, the Ethics Committee granted a waiver of informed consent from the patients.

## Supporting information


Figure S1.

Figure S2.

Figure S3.

Table S1.

Table S2.

Table S3.

## Data Availability

All supporting data can be obtained from the corresponding author upon reasonable request.
