# Supplementary figures and images for: Association of immune checkpoint inhibitors therapy with arterial thromboembolic events in cancer patients: A retrospective cohort study
Source: Cancer Med. 2023 Aug 16;12(18):18531–41. doi: 10.1002/cam4.6455 (PMC10557854; doi:10.1002/cam4.6455)

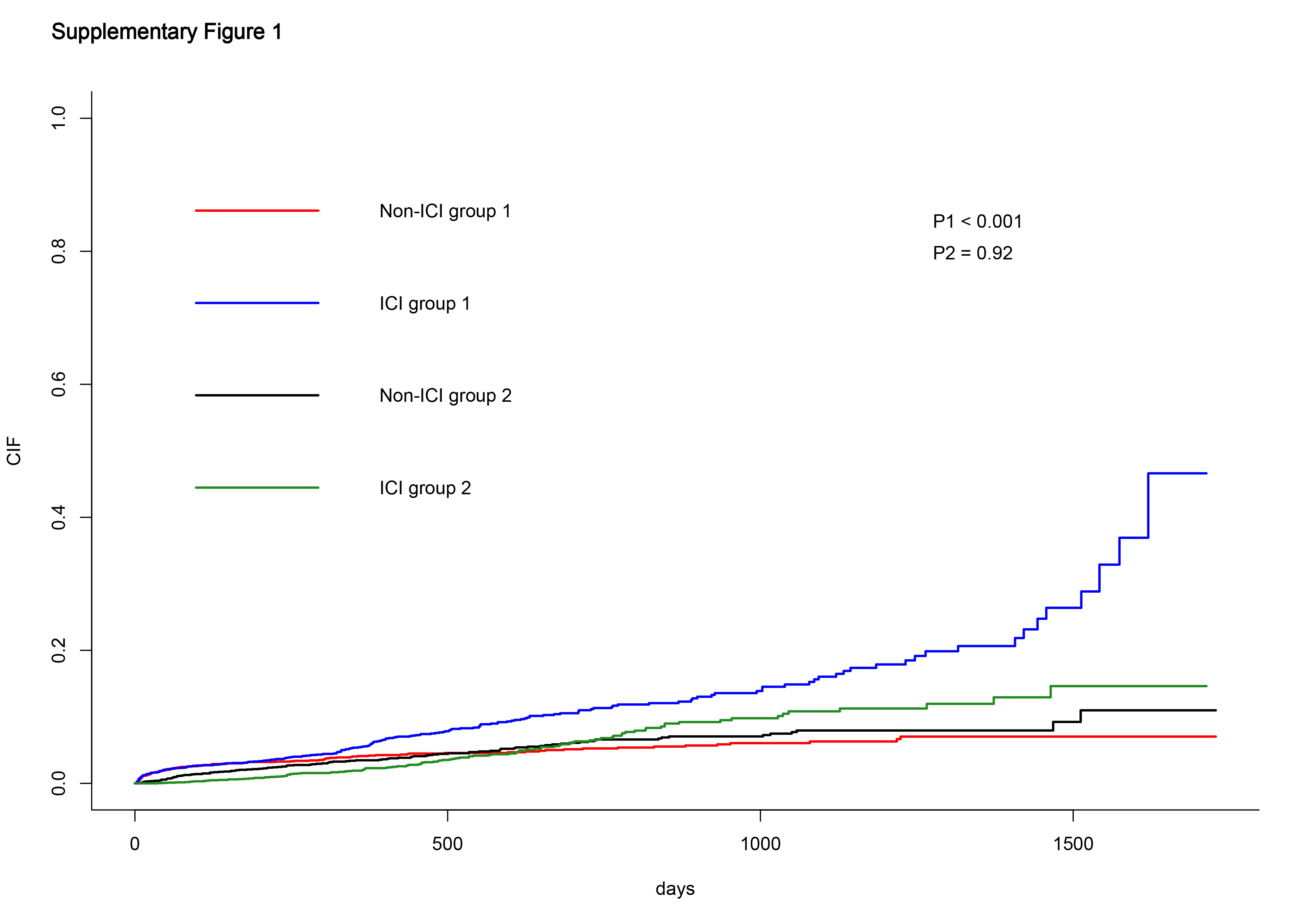

Supplement: Supplementary file 1 — Figure S1. [file CAM4-12-18531-s002.tif]

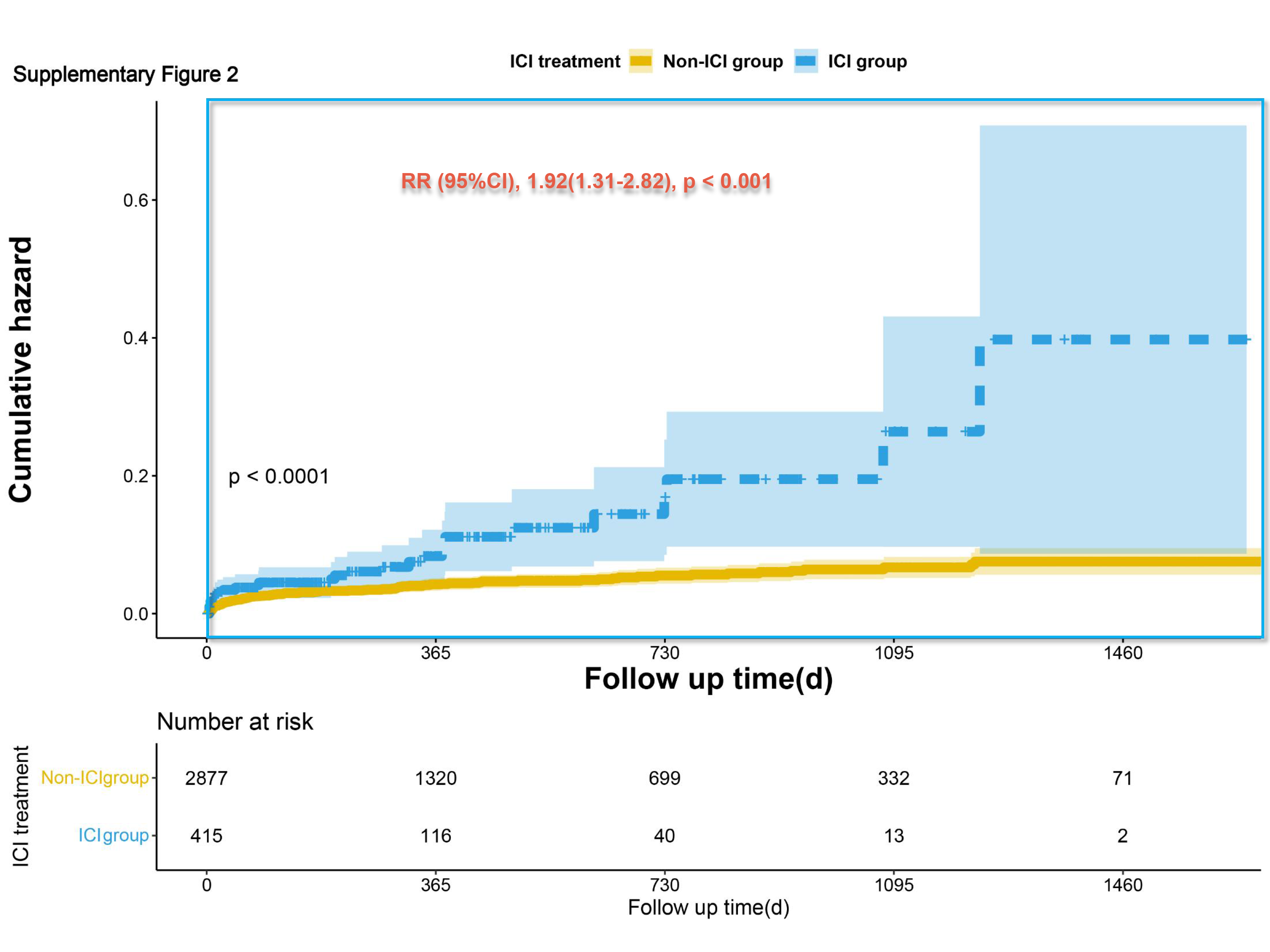

Supplement: Supplementary file 2 — Figure S2. [file CAM4-12-18531-s005.tif]

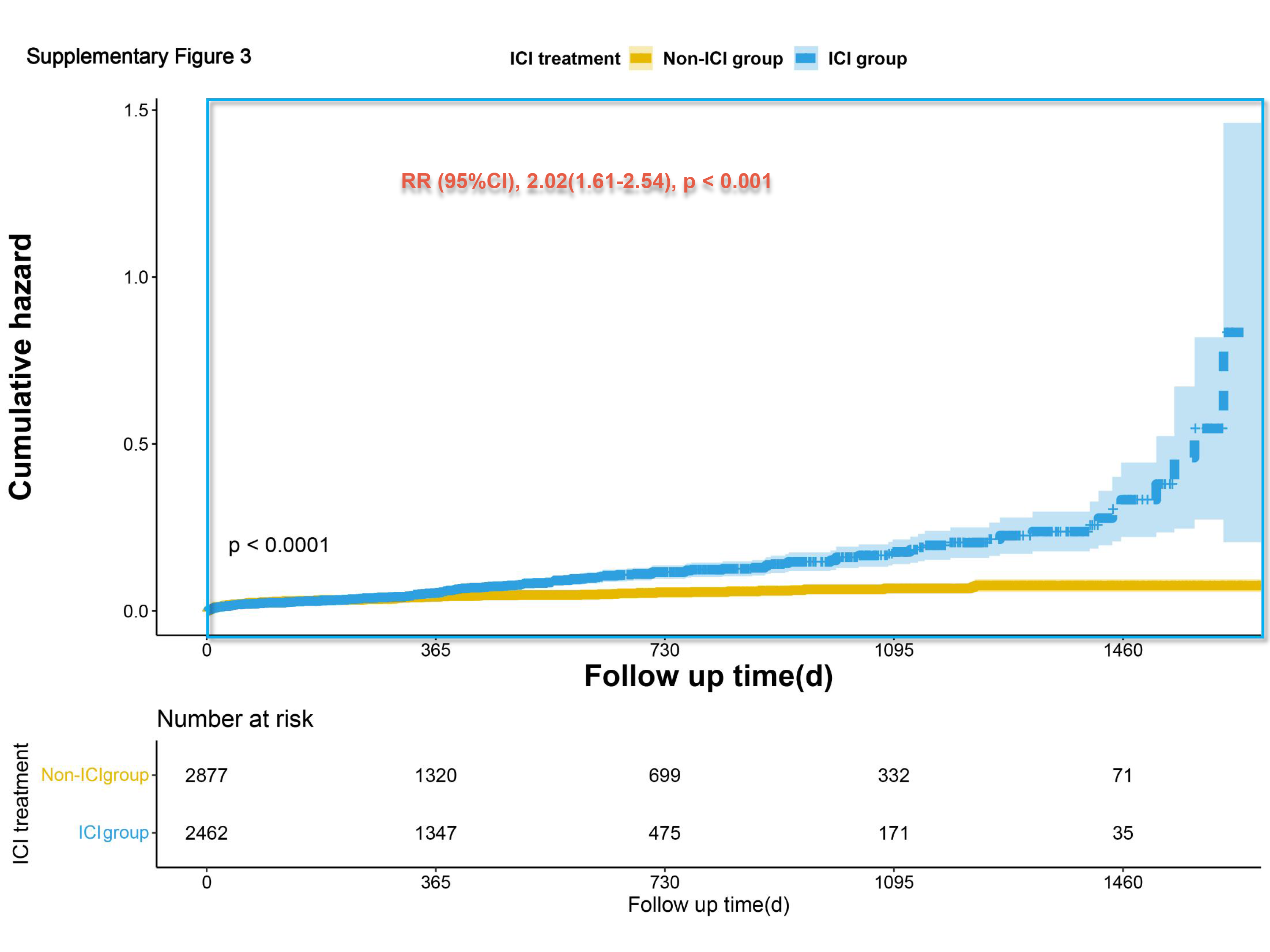

Supplement: Supplementary file 3 — Figure S3. [file CAM4-12-18531-s006.tif]
