# Supplementary material for: Association of immune checkpoint inhibitors therapy with arterial thromboembolic events in cancer patients: A retrospective cohort study
Source: Cancer Med. 2023 Aug 16;12(18):18531–41. doi: 10.1002/cam4.6455 (PMC10557854; doi:10.1002/cam4.6455)
Supplement: Supplementary file 4 — Table S1. [file CAM4-12-18531-s004.docx]

**Supplementary Table 1: ICD-10 Codes for Acute Coronary Syndrome**

| ***Acute coronary syndrome*** | |
| --- | --- |
| **ICD-10 codes** | **Nomenclature of disease** |
| I20.0 | |
| I20.000 | Unstable angina |
| I20.001 | Crescendo angina |
| I20.002 | De novo effort angina |
| I20.003 | Worsening effort angina |
| I20.004 | Supine angina |
| I20.005 | Intermediate coronary syndrome |
| I20.006 | Preinfarction syndrome |
| I20.1 | |
| I20.100 | Angina pectoris with documented spasm |
| I20.101 | Angiospastic angina |
| I20.102 | Prinzmetal angina |
| I20.103 | Spasm-induced angina |
| I20.104 | Variant angina |
| I21 | |
| I21.000 | ST elevation (STEMI) myocardial infarction of anterior wall |
| I21.001 | ST elevation (STEMI) myocardial infarction involving left main coronary artery |
| I21.002 | ST elevation (STEMI) myocardial infarction involving left anterior descending coronary artery |
| I21.003 | ST elevation (STEMI) myocardial infarction involving other coronary artery of anterior wall |
| I21.100 | ST elevation (STEMI) myocardial infarction of inferior wall |
| I21.101 | ST elevation (STEMI) myocardial infarction involving right coronary artery |
| I21.102 | ST elevation (STEMI) myocardial infarction involving other coronary artery of inferior wall |
| I21.200 | ST elevation (STEMI) myocardial infarction of other sites |
| I21.201 | ST elevation (STEMI) myocardial infarction involving left circumflex coronary artery |
| I21.209 | ST elevation (STEMI) myocardial infarction involving other sites |
| I21.300 | ST elevation (STEMI) myocardial infarction of unspecified site |
| I21.400 | Non-ST elevation (NSTEMI) myocardial infarction |
| I21.900 | Acute myocardial infarction, unspecified |
| I22 | |
| I22.000 | Subsequent ST elevation (STEMI) myocardial infarction of anterior wall |
| I22.100 | Subsequent ST elevation (STEMI) myocardial infarction of inferior wall |
| I22.200 | Subsequent non-ST elevation (NSTEMI) myocardial infarction |
| I22.800 | Subsequent ST elevation (STEMI) myocardial infarction of other sites |
| I22.900 | Subsequent ST elevation (STEMI) myocardial infarction of unspecified site |
| I23 | |
| I23.000 | Hemopericardium as current complication following acute myocardial infarction |
| I23.100 | Atrial septal defect as current complication following acute myocardial infarction |
| I23.200 | Ventricular septal defect as current complication following acute myocardial infarction |
| I23.300 | Rupture of cardiac wall without hemopericardium as current complication following acute myocardial infarction |
| I23.400 | Rupture of chordae tendineae as current complication following acute myocardial infarction |
| I23.500 | Rupture of papillary muscle as current complication following acute myocardial infarction |
| I23.600 | Thrombosis of atrium, auricular appendage, and ventricle as current complications following acute myocardial infarction |
| I23.700 | Postinfarction angina |
| I23.800 | Other current complications following acute myocardial infarction |
