# Supplementary material for: Association of immune checkpoint inhibitors therapy with arterial thromboembolic events in cancer patients: A retrospective cohort study
Source: Cancer Med. 2023 Aug 16;12(18):18531–41. doi: 10.1002/cam4.6455 (PMC10557854; doi:10.1002/cam4.6455)
Supplement: Supplementary file 5 — Table S2. [file CAM4-12-18531-s003.docx]

**Supplementary Table 2: ICD-10 Codes for Stroke/Transient Ischemic Attack**

| ***Stroke/Transient ischemic attack*** | |
| --- | --- |
| **ICD-10 codes** | **Nomenclature of disease** |
| I63.0 | |
| I63.000 | Cerebral infarction due to thrombosis of unspecified precerebral artery |
| I63.010 | Cerebral infarction due to thrombosis of vertebral artery |
| I63.011 | Cerebral infarction due to thrombosis of right vertebral artery |
| I63.012 | Cerebral infarction due to thrombosis of left vertebral artery |
| I63.013 | Cerebral infarction due to thrombosis of bilateral vertebral arteries |
| I63.019 | Cerebral infarction due to thrombosis of unspecified vertebral artery |
| I63.020 | Cerebral infarction due to thrombosis of basilar artery |
| I63.030 | Cerebral infarction due to thrombosis of carotid artery |
| I63.031 | Cerebral infarction due to thrombosis of right carotid artery |
| I63.032 | Cerebral infarction due to thrombosis of left carotid artery |
| I63.033 | Cerebral infarction due to thrombosis of bilateral carotid arteries |
| I63.039 | Cerebral infarction due to thrombosis of unspecified carotid artery |
| I63.090 | Cerebral infarction due to thrombosis of other precerebral artery |
| I63.1 | |
| I63.100 | Cerebral infarction due to embolism of unspecified precerebral artery |
| I63.110 | Cerebral infarction due to embolism of vertebral artery |
| I63.111 | Cerebral infarction due to embolism of right vertebral artery |
| I63.112 | Cerebral infarction due to embolism of left vertebral artery |
| I63.113 | Cerebral infarction due to embolism of bilateral vertebral arteries |
| I63.119 | Cerebral infarction due to embolism of unspecified vertebral artery |
| I63.120 | Cerebral infarction due to embolism of basilar artery |
| I63.130 | Cerebral infarction due to embolism of carotid artery |
| I63.131 | Cerebral infarction due to embolism of right carotid artery |
| I63.132 | Cerebral infarction due to embolism of left carotid artery |
| I63.133 | Cerebral infarction due to embolism of bilateral carotid arteries |
| I63.139 | Cerebral infarction due to embolism of unspecified carotid artery |
| I63.190 | Cerebral infarction due to embolism of other precerebral artery |
| I63.2 | |
| I63.200 | Cerebral infarction due to unspecified occlusion or stenosis of unspecified precerebral arteries |
| I63.210 | Cerebral infarction due to unspecified occlusion or stenosis of vertebral arteries |
| I63.211 | Cerebral infarction due to unspecified occlusion or stenosis of right vertebral artery |
| I63.212 | Cerebral infarction due to unspecified occlusion or stenosis of left vertebral artery |
| I63.213 | Cerebral infarction due to unspecified occlusion or stenosis of bilateral vertebral arteries |
| I63.219 | Cerebral infarction due to unspecified occlusion or stenosis of unspecified vertebral artery |
| I63.220 | Cerebral infarction due to unspecified occlusion or stenosis of basilar artery |
| I63.230 | Cerebral infarction due to unspecified occlusion or stenosis of carotid arteries |
| I63.231 | Cerebral infarction due to unspecified occlusion or stenosis of right carotid arteries |
| I63.232 | Cerebral infarction due to unspecified occlusion or stenosis of left carotid arteries |
| I63.233 | Cerebral infarction due to unspecified occlusion or stenosis of bilateral carotid arteries |
| I63.239 | Cerebral infarction due to unspecified occlusion or stenosis of unspecified carotid artery |
| I63.290 | Cerebral infarction due to unspecified occlusion or stenosis of other precerebral arteries |
| I63.3 | |
| I63.300 | Cerebral infarction due to thrombosis of unspecified cerebral artery |
| I63.310 | Cerebral infarction due to thrombosis of middle cerebral artery |
| I63.311 | Cerebral infarction due to thrombosis of right middle cerebral artery |
| I63.312 | Cerebral infarction due to thrombosis of left middle cerebral artery |
| I63.313 | Cerebral infarction due to thrombosis of bilateral middle cerebral arteries |
| I63.319 | Cerebral infarction due to thrombosis of unspecified middle cerebral artery |
| I63.320 | Cerebral infarction due to thrombosis of anterior cerebral artery |
| I63.321 | Cerebral infarction due to thrombosis of right anterior cerebral artery |
| I63.322 | Cerebral infarction due to thrombosis of left anterior cerebral artery |
| I63.323 | Cerebral infarction due to thrombosis of bilateral anterior cerebral arteries |
| I63.329 | Cerebral infarction due to thrombosis of unspecified anterior cerebral artery |
| I63.330 | Cerebral infarction due to thrombosis of posterior cerebral artery |
| I63.331 | Cerebral infarction due to thrombosis of right posterior cerebral artery |
| I63.332 | Cerebral infarction due to thrombosis of left posterior cerebral artery |
| I63.333 | Cerebral infarction due to thrombosis of bilateral posterior cerebral arteries |
| I63.339 | Cerebral infarction due to thrombosis of unspecified posterior cerebral artery |
| I63.340 | Cerebral infarction due to thrombosis of cerebellar artery |
| I63.341 | Cerebral infarction due to thrombosis of right cerebellar artery |
| I63.342 | Cerebral infarction due to thrombosis of left cerebellar artery |
| I63.343 | Cerebral infarction due to thrombosis of bilateral cerebellar arteries |
| I63.349 | Cerebral infarction due to thrombosis of unspecified cerebellar artery |
| I63.390 | Cerebral infarction due to thrombosis of other cerebral artery |
| I63.4 | |
| I63.400 | Cerebral infarction due to embolism of unspecified cerebral artery |
| I63.410 | Cerebral infarction due to embolism of middle cerebral artery |
| I63.411 | Cerebral infarction due to embolism of right middle cerebral artery |
| I63.412 | Cerebral infarction due to embolism of left middle cerebral artery |
| I63.413 | Cerebral infarction due to embolism of bilateral middle cerebral arteries |
| I63.419 | Cerebral infarction due to embolism of unspecified middle cerebral artery |
| I63.420 | Cerebral infarction due to embolism of anterior cerebral artery |
| I63.421 | Cerebral infarction due to embolism of right anterior cerebral artery |
| I63.422 | Cerebral infarction due to embolism of left anterior cerebral artery |
| I63.423 | Cerebral infarction due to embolism of bilateral anterior cerebral arteries |
| I63.429 | Cerebral infarction due to embolism of unspecified anterior cerebral artery |
| I63.430 | Cerebral infarction due to embolism of posterior cerebral artery |
| I63.431 | Cerebral infarction due to embolism of right posterior cerebral artery |
| I63.432 | Cerebral infarction due to embolism of left posterior cerebral artery |
| I63.433 | Cerebral infarction due to embolism of bilateral posterior cerebral arteries |
| I63.439 | Cerebral infarction due to embolism of unspecified posterior cerebral artery |
| I63.440 | Cerebral infarction due to embolism of cerebellar artery |
| I63.441 | Cerebral infarction due to embolism of right cerebellar artery |
| I63.442 | Cerebral infarction due to embolism of left cerebellar artery |
| I63.443 | Cerebral infarction due to embolism of bilateral cerebellar arteries |
| I63.449 | Cerebral infarction due to embolism of unspecified cerebellar artery |
| I63.490 | Cerebral infarction due to embolism of other cerebral artery |
| I63.5 | |
| I63.500 | Cerebral infarction due to unspecified occlusion or stenosis of unspecified cerebral artery |
| I63.510 | Cerebral infarction due to unspecified occlusion or stenosis of middle cerebral artery |
| I63.511 | Cerebral infarction due to unspecified occlusion or stenosis of right middle cerebral artery |
| I63.512 | Cerebral infarction due to unspecified occlusion or stenosis of left middle cerebral artery |
| I63.513 | Cerebral infarction due to unspecified occlusion or stenosis of bilateral middle cerebral arteries |
| I63.519 | Cerebral infarction due to unspecified occlusion or stenosis of unspecified middle cerebral artery |
| I63.520 | Cerebral infarction due to unspecified occlusion or stenosis of anterior cerebral artery |
| I63.521 | Cerebral infarction due to unspecified occlusion or stenosis of right anterior cerebral artery |
| I63.522 | Cerebral infarction due to unspecified occlusion or stenosis of left anterior cerebral artery |
| I63.523 | Cerebral infarction due to unspecified occlusion or stenosis of bilateral anterior cerebral arteries |
| I63.529 | Cerebral infarction due to unspecified occlusion or stenosis of unspecified anterior cerebral artery |
| I63.530 | Cerebral infarction due to unspecified occlusion or stenosis of posterior cerebral artery |
| I63.531 | Cerebral infarction due to unspecified occlusion or stenosis of right posterior cerebral artery |
| I63.532 | Cerebral infarction due to unspecified occlusion or stenosis of left posterior cerebral artery |
| I63.533 | Cerebral infarction due to unspecified occlusion or stenosis of bilateral posterior cerebral arteries |
| I63.539 | Cerebral infarction due to unspecified occlusion or stenosis of unspecified posterior cerebral artery |
| I63.540 | Cerebral infarction due to unspecified occlusion or stenosis of cerebellar artery |
| I63.541 | Cerebral infarction due to unspecified occlusion or stenosis of right cerebellar artery |
| I63.542 | Cerebral infarction due to unspecified occlusion or stenosis of left cerebellar artery |
| I63.543 | Cerebral infarction due to unspecified occlusion or stenosis of bilateral cerebellar arteries |
| I63.549 | Cerebral infarction due to unspecified occlusion or stenosis of unspecified cerebellar artery |
| I63.590 | Cerebral infarction due to unspecified occlusion or stenosis of other cerebral artery |
| I63.6 | |
| I63.600 | Cerebral infarction due to occlusion or stenosis of small artery |
| I63.800 | Cerebral infarction due to cerebral venous thrombosis, nonpyogenic |
| I63.9 | |
| I63.900 | Cerebral infarction, unspecified |
| G45.900 | |
| G45.900 | Transient ischemic attack |
