# Supplementary material for: Association of immune checkpoint inhibitors therapy with arterial thromboembolic events in cancer patients: A retrospective cohort study
Source: Cancer Med. 2023 Aug 16;12(18):18531–41. doi: 10.1002/cam4.6455 (PMC10557854; doi:10.1002/cam4.6455)
Supplement: Supplementary file 6 — Table S3. [file CAM4-12-18531-s001.docx]

**Supplementary Table 3: ICD-10 Codes for Peripheral arterial thromboembolism**

| ***Peripheral arterial thromboembolism*** | |
| --- | --- |
| **ICD-10 codes** | **Nomenclature of disease** |
| I74.0 | |
| I74.000 | Embolism and thrombosis of abdominal aorta |
| I74.001 | Saddle embolus of abdominal aorta |
| I74.009 | Other arterial embolism and thrombosis of abdominal aorta |
| I74.1 | |
| I74.100 | Embolism and thrombosis of other and unspecified parts of aorta |
| I74.101 | Embolism and thrombosis of unspecified parts of aorta |
| I74.102 | Embolism and thrombosis of thoracic aorta |
| I74.109 | Embolism and thrombosis of other parts of aorta |
| I74.2 | |
| I74.200 | Embolism and thrombosis of arteries of the upper extremities |
| I74.3 | |
| I74.300 | Embolism and thrombosis of arteries of the lower extremities |
| I74.4 | |
| I74.400 | Embolism and thrombosis of arteries of extremities, unspecified |
| I74.5 | |
| I74.500 | Embolism and thrombosis of iliac artery |
| I74.8 | |
| I74.800 | Embolism and thrombosis of other arteries |
| I74.9 | |
| I74.900 | Embolism and thrombosis of unspecified artery |
| K55.003 | |
| K55.003 | Embolism and thrombosis of the mesenteric arteries |
| N28.0 | |
| N28.000 | Renal infarction |
| N28.001 | Renal artery occlusion |
| N28.002 | Embolism and thrombosis of renal arteries |
| D73.500 | |
| D73.500 | Splenic infarction |
